# Groundwater vulnerability to pollution mapping of Ranchi district using GIS

**DOI:** 10.1007/s13201-014-0198-2

**Published:** 2014-05-17

**Authors:** R. Krishna, J. Iqbal, A. K. Gorai, G. Pathak, F. Tuluri, P. B. Tchounwou

**Affiliations:** Environmental Science and Engineering Group, Birla Institute of Technology Mesra, Ranchi 835215, India; Environmental Science and Engineering Group, Birla Institute of Technology Mesra, Ranchi 835215, India; Environmental Science and Engineering Group, Birla Institute of Technology Mesra, Ranchi 835215, India; Environmental Science and Engineering Group, Birla Institute of Technology Mesra, Ranchi 835215, India; Department of Technology, Jackson State University, Jackson, MS 39217, USA; Department of Biology, Jackson State University, Jackson, MS 39217, USA

**Keywords:** Groundwater vulnerability, Ranchi, DRASTIC, Sensitivity analysis, GIS

## Abstract

Groundwater pollution due to anthropogenic activities is one of the major environmental problems in urban and industrial areas. The present study demonstrates the integrated approach with GIS and DRASTIC model to derive a groundwater vulnerability to pollution map. The model considers the seven hydrogeological factors [Depth to water table (*D*), net recharge (*R*), aquifer media (*A*), soil media (*S*), topography or slope (*T*), impact of vadose zone (*I*) and hydraulic Conductivity(*C*)] for generating the groundwater vulnerability to pollution map. The model was applied for assessing the groundwater vulnerability to pollution in Ranchi district, Jharkhand, India. The model was validated by comparing the model output (vulnerability indices) with the observed nitrate concentrations in groundwater in the study area. The reason behind the selection of nitrate is that the major sources of nitrate in groundwater are anthropogenic in nature. Groundwater samples were collected from 30 wells/tube wells distributed in the study area. The samples were analyzed in the laboratory for measuring the nitrate concentrations in groundwater. A sensitivity analysis of the integrated model was performed to evaluate the influence of single parameters on groundwater vulnerability index. New weights were computed for each input parameters to understand the influence of individual hydrogeological factors in vulnerability indices in the study area. Aquifer vulnerability maps generated in this study can be used for environmental planning and groundwater management.

## Introduction

Groundwater is the most important water resource on earth (Villeneuve et al. 1990). Groundwater quality is under considerable threat of contamination especially in agriculture-dominated areas due to intense use of fertilizers and pesticides (Giambelluca et al. 1996; Soutter and Musy 1998; Lake et al. 2003; Thapinta and Hudak 2003; Chae et al. 2004). Thus, the protection of groundwater against anthropogenic pollution is of crucial importance (Zektser et al. 2004). Assessment of groundwater vulnerability to pollution helps to determine the proneness of groundwater contamination and hence essential for managing and preserving the groundwater quality (Fobe and Goossens 1990; Worrall et al. 2002; Worrall and Besien 2004).

Groundwater vulnerability to pollution studies helps to categorize the land on the basis of its proneness to vulnerability (Gogu and Dassargues 2000a). That is, ground-water vulnerability assessment delineates areas that are more susceptible to contamination on the basis of the different hydrogeological factors and anthropogenic sources. In general, the study explains the estimation of the contaminants migration potential from land surface to groundwater through the unsaturated zones (Connell and Daele 2003). Groundwater vulnerability assessment is essential for management of groundwater resources and subsequent land use planning (Rupert 2001; Babiker et al. 2005). Groundwater vulnerability maps provide visual information for more vulnerable zones which help to protect groundwater resources and also to evaluate the potential for water quality improvement by changing the agricultural practices and land use applications (Connell and Daele 2003; Rupert 2001; Babiker et al. 2005; Burkart and Feher 1996).

Groundwater vulnerability assessment can be used in planning, policy analysis, and decision making, viz., advising decision makers for adopting specific management options to mitigate the quality of groundwater resources; demonstrating the implications and consequences of their decisions; providing direction for using groundwater resources; highlighting about proper land use practices and activities; and educating the general public regarding the consequences of groundwater contamination (NRC 1993).

The concept of aquifer vulnerability to external pollution was introduced in 1960s by (Margat 1968), with several systems of aquifer vulnerability assessment developed in the following years (Aller et al. 1987; Civita 1994; Vrba and Zaporozec 1994; Sinan and Razack 2009; Polemio et al. 2009; Foster 1987). They found that the reason behind the different vulnerabilities is the different hydrogeological settings. Many approaches have been developed to evaluate aquifer vulnerability. These are overlay/index methods, process-based methods and statistical methods (Zhang et al. 1996; Tesoriero et al. 1998). The overlay/index methods use location-specific vulnerability indices based on the hydrogeological factors controlling movement of pollutants from the land surface to the water bearing strata. The process-based methods use contaminants transport models to estimate the contaminant migration (Barbash and Resek 1996). Statistical methods estimate the associations between the spatial variables and the occurrence of pollutants in the groundwater using various statistics.

Among all the approaches mentioned above, the overlay and index method has been the most widely adopted approach for wide-scale groundwater vulnerability assessments. Scientist started giving predictions of groundwater pollution potential based on hydrogeological settings (Polemio et al. 2009; Almasri Mohammad 2008; Berkhoff 2008; Rahman 2008; Massone et al. 2010; Kwansiririkul et al. 2004; Kim and Hamm 1999; Secunda et al. 1998; Vias et al. 2005; Brosig Karolin et al. 2008; Ferreira Lobo and Oliveira Manuel 2004; Nouri and Malmasi 2005; Herlinger and Viero 2007; Shirazi et al. 2012, 2013; Neshat Aminreza et al. 2014). This paper aims to demonstrate a GIS-based DRASTIC model for groundwater vulnerability assessment of Ranchi district. The validation of the model prediction was done on the basis of observed nitrate concentration in groundwater in the study area. Sensitivity analysis of the model was also carried out to understand the influence of the individual input variables on groundwater vulnerability to pollution index.

## Study area

**Fig. 1:**
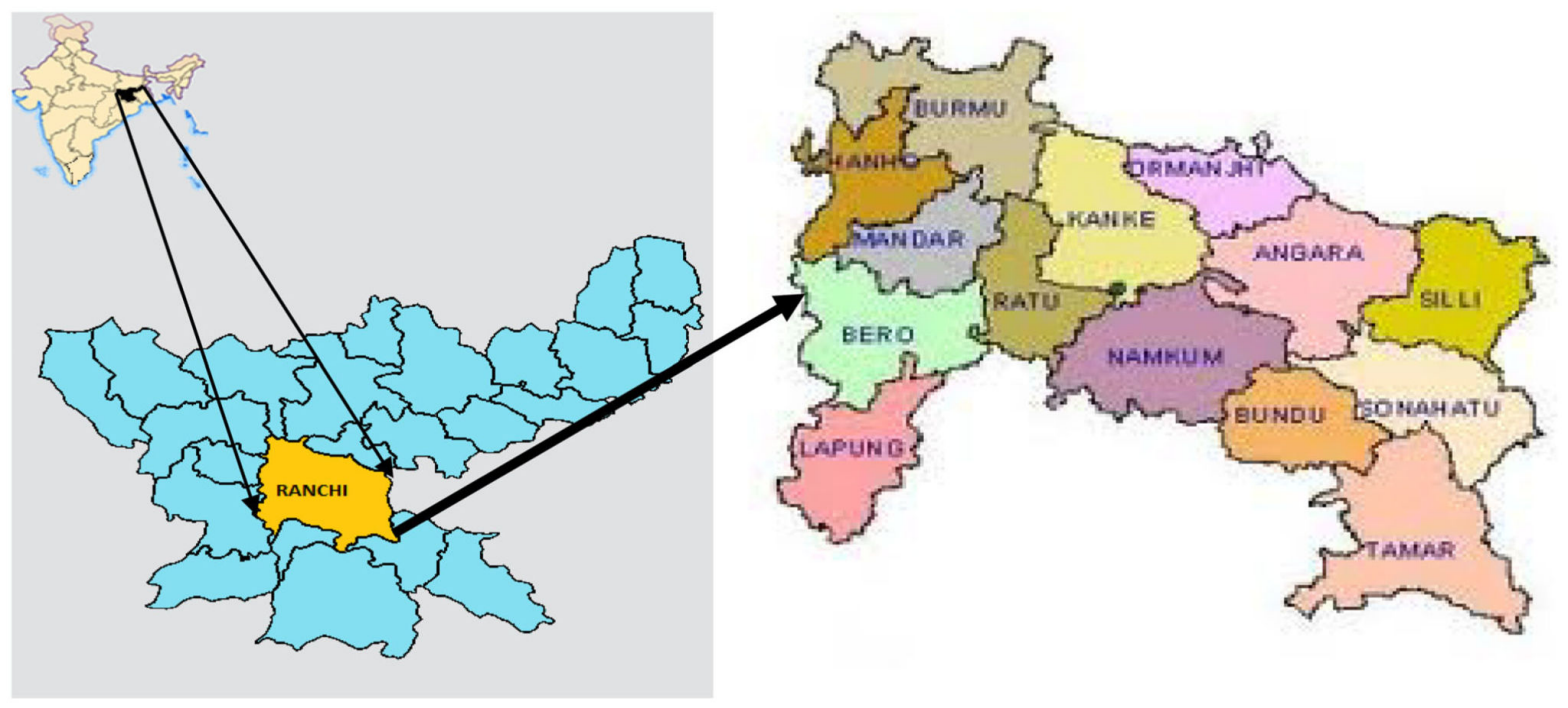
Study area map

The area selected for the proposed study is Ranchi district. Ranchi district lies in the southern part of Jharkhand state and bounded by other district of Jharkhand, viz., Hazaribagh, West Singhbhum, Gumla, Lohardaga, and East Singhbhum. This is also bounded by Purulia district of West Bengal. The district has a total area of 4,912 km^2^ and is located between 22°45′–23°45′ North latitude to 84°45′–84°50′ East longitude. The district comprises of 14 blocks namely Ormanjhi, Kanke, Ratu, Bero, Burmu, Lapung, Chanho, Mandar, Bundu, Tamar, Angara, Sonahatu, Silli, Namkum as shown in Fig. 1.

The climate of Ranchi district is a subtropical climate. This is characterized by hot summer season from March to May and well-distributed rainfall during southwest monsoon season from June to October. Ranchi district has varied hydrogeological characteristics and hence the groundwater potential differs from one location to another. The three-fourth of the district area is underlain by Chotanagpur granite gneiss of pre-Cambrian age (CGWB 2009). In two blocks (Ratu and Bero) thick lateritic capping is placed above granite gneiss. A big patch of older alluvium exists in Mandar block and limestone rock exists in northernmost portion of Burmu block. The northernmost and southernmost parts of the district are mainly covered with hillocks and forests. In general, the altitude of the area varies from 500 to 700 m above mean sea level, but there are many hillocks through the district having altitude more than 700 m above mean sea level. Two types of aquifers (Weathered aquifer and fractured aquifers) exist in the study area. Thickness of weathered aquifers varies from 10 to 25 m in granite terrain and 30 to 60 m in lateritic terrain. In weathered aquifers groundwater occurs in unconfined condition, while in fractured aquifer groundwater occurs in semi-confined to confined conditions.

## Materials and methods

**Fig. 2:**
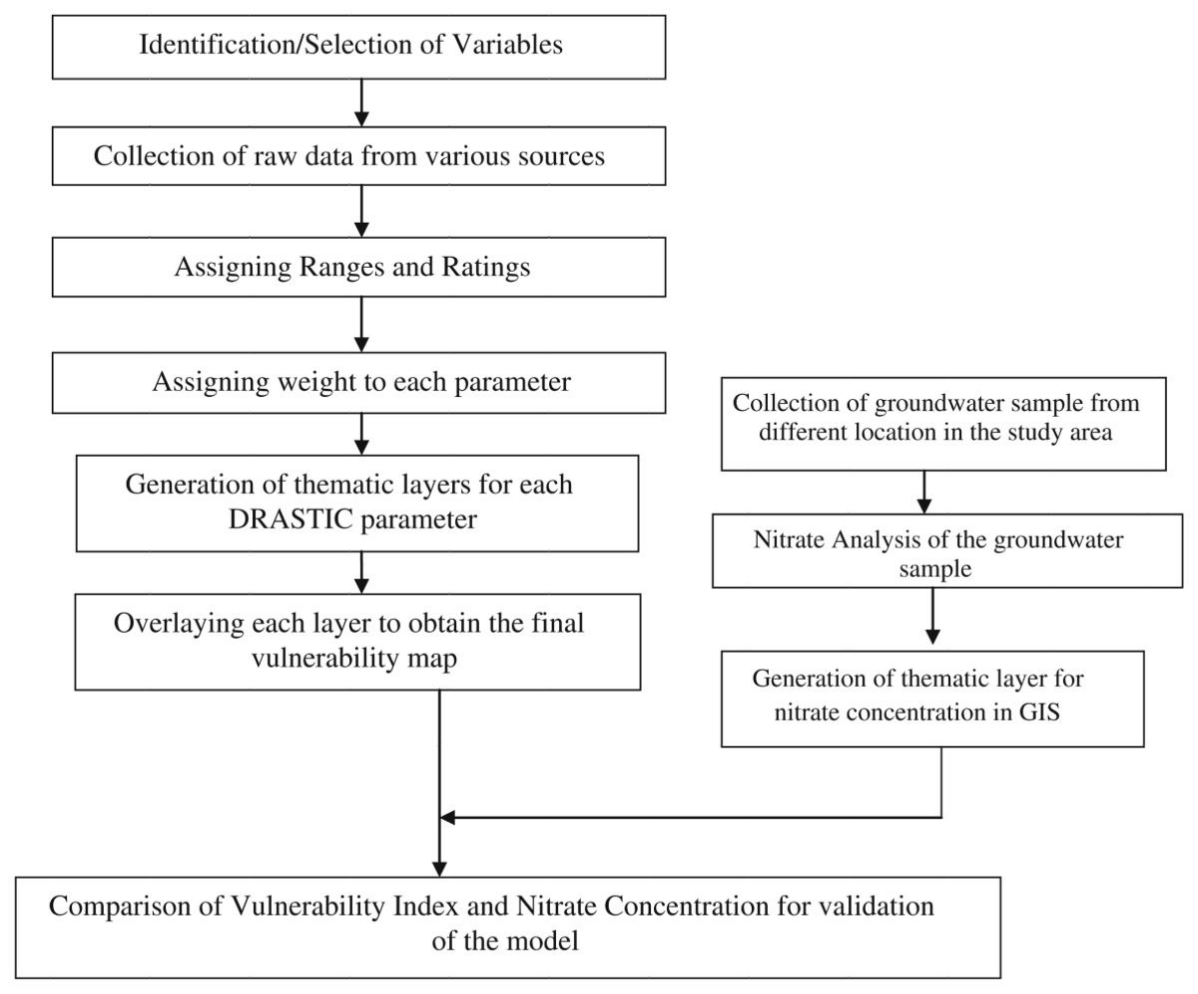
Flow chart of the working methodology

Groundwater vulnerability was evaluated using hydrogeological factors that can influence the pollutant transport through the vadose zone to the water bearing strata using GIS-based DRASTIC (Aller et al. 1987) method. The flowchart (shown in Fig. 2) represents the general overview of the research methodology. In the present study, seven hydrogeological parameters [Depth to water table (*D*), net recharge (*R*), aquifer media (*A*), soil media (*S*), topography (*T*), impact of vadose zone (*I*) and hydraulic Conductivity (*C*)] were considered for assessing the groundwater vulnerability.

Thematic maps of seven factors (*D*, *R*, *A*, *S*, *T*, *I*, and *C*) were generated and used for producing the final ground-water vulnerability to pollution index map. The thematic values in each of the seven hydrogeological maps were classified into corresponding ranges as per the DRASTIC model. Each range was assigned their corresponding ratings as per the DRASTIC model. Weight multipliers were then used for each factor to balance and enhance its importance. The final vulnerability map was computed as the weighted sum overlay of the seven layers using Eq. (1) and was termed as DRASTIC INDEX (DI).

(1) $$\text{DRASTIC.INDEX}\;(\text{DI})=D_{r}D_{w}+R_{r}R_{w}+A_{r}A_{w}+S_{r}S_{w}+T_{r}T_{w}+I_{r}I_{w}+C_{r}C_{w}$$

where, *D*_r_, *R*_r_, *A*_r_, *S*_r_, *T*_r_, *I*_r_, and *C*_r_ are ratings assigned to depth to water table, net recharge, aquifer media, soil media, topography or slope, impact of vadose zone, and hydraulic conductivity, respectively.

*D*_w_, *R*_w_, *A*_w_, *S*_w_, *T*_w_, *I*_w_, and *C*_w_ are weights assigned to depth to water table, net recharge, aquifer media, soil media, topography or slope, impact of vadose zone, and hydraulic conductivity, respectively.

Every parameter in the model assigned a fixed weight (listed in Table 1) indicating the relative influence of the parameter in transporting contaminants to the groundwater. Each input factor has been divided into either ranges or significant media types that affect groundwater vulnerability. The media types such as aquifer material, soil type and impact of vadose zone, cannot be measured numerically and hence ratings were assigned to each type of media. Each range of each DRASTIC parameter has been evaluated with respect to the others to determine its relative significance to pollution potential, and has been assigned a rating of 1–10. The “easiest to be polluted” was assigned a rating ten, except net recharge (which is 9) and the “most difficult to pollute” was assigned a rating of one. The numerical ratings, which were established using the Delphi technique (Aller et al. 1987), are well defined and have been used worldwide (Al-Adamat et al. 2003; Anwar et al. 2003; Chandrashekhar et al. 1999; Dixon 2005). The ratings for each parameter are listed in Table 1 for all the ranges and types.

## Data sources and generation of thematic layers

The raw data were collected or derived from various published reports/maps for the generation of the thematic layers and are listed in Table 2. Thematic layers for each hydrogeological parameter were generated using ArcGIS software version 9.3.

### Depth to groundwater

**Fig. 3:**
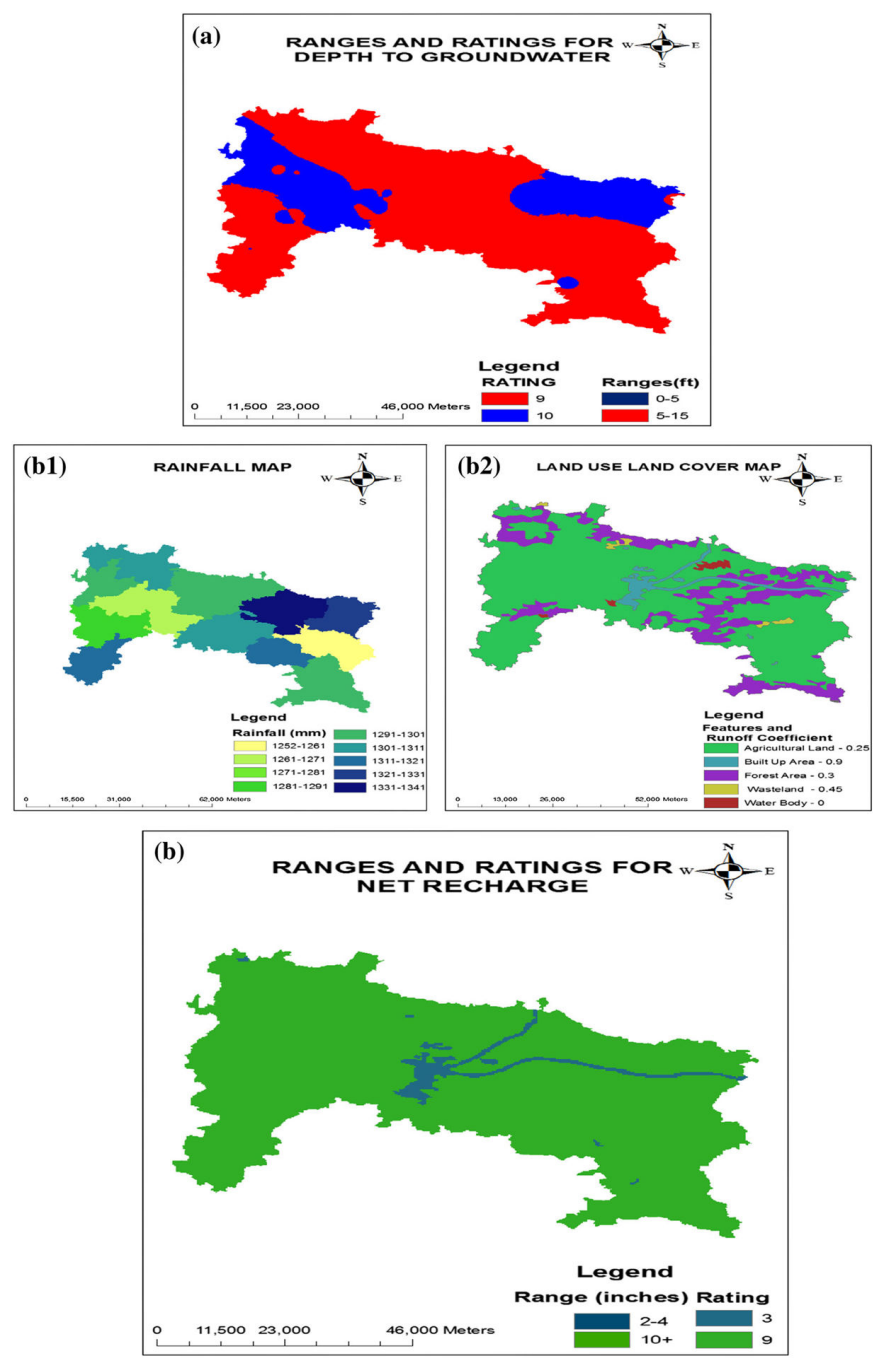

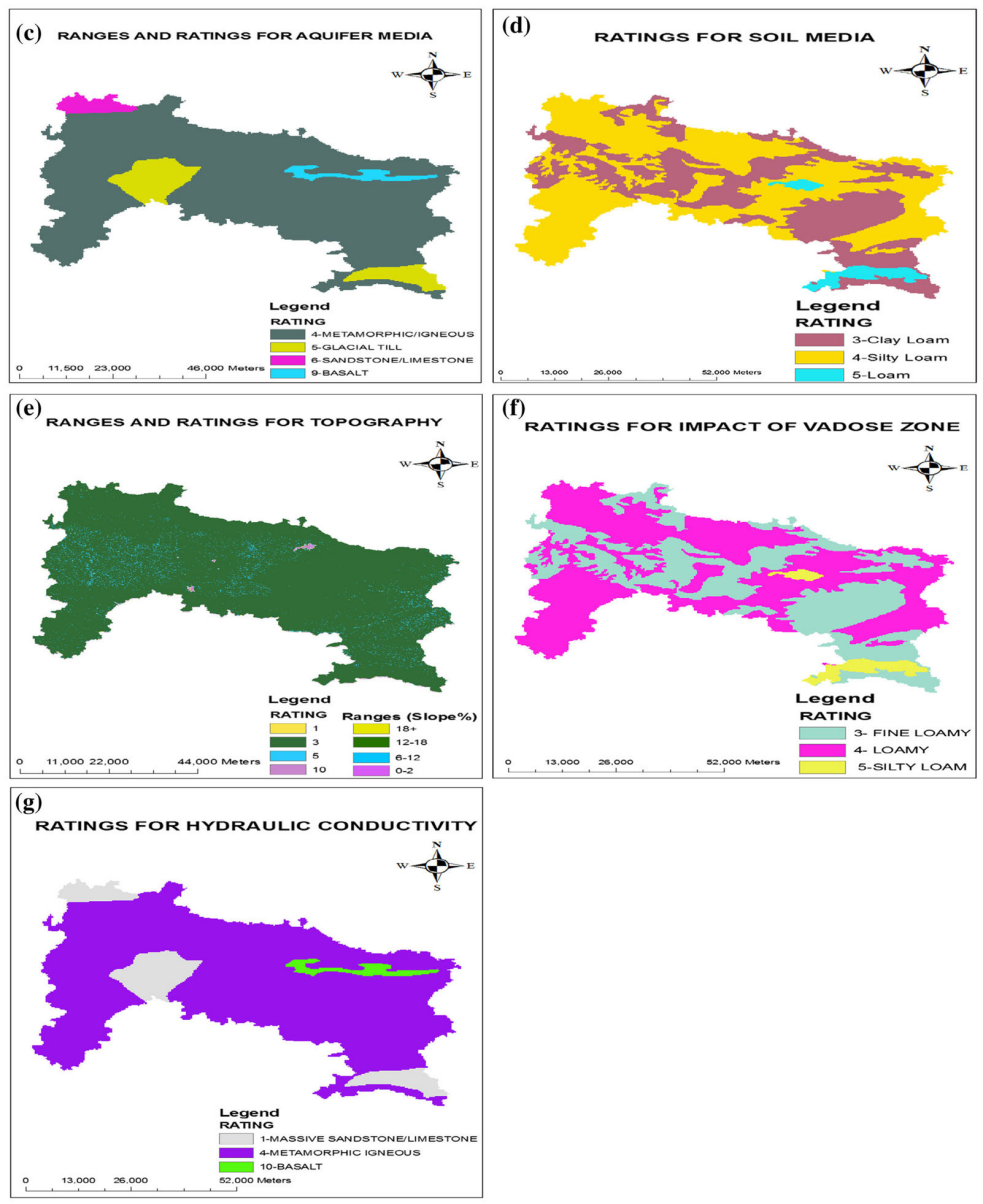
**a** Depth to groundwater, **b1** rainfall map, **b2** land use map, **b** net recharge, **c** aquifer media, **d** soil media, **e** topography, **f** impact of vadose zone, **g** hydraulic Conductivity

The depth to groundwater table parameter was derived from water level data collected from Central Ground Water Board (CGWB), Ranchi. The depth to groundwater table is shallow and has a range of 2–15 ft below ground level. The well data were then used to generate the map for depth to water table contoured by interpolating using inverse distance weighted (IDW) method. The study area was extracted using the district boundary as a mask. The thematic map was reclassified into two classes, corresponds to the DRASTIC model range value (listed in Table 1). Though, the ranges defined for different classes (in Table 1) are in meter, these values were converted into feet during rating assignment. The depth to water table values and their corresponding ratings are shown in Table 3. The map generated for depth to water is shown in Fig. 3a.

### Net recharge

The thematic map of precipitation was generated using the rainfall data collected from Indian Meteorological Division (IMD), India as shown in Fig. 3b1. The evapotranspiration map was derived from precipitation map by assuming the rate of evapotranspiration to be 5 % of the precipitation (value taken from a report of IMD, Ranchi). The land use map of the study area was prepared and reclassified into five categories as agricultural land, built-up area, forest area, waste land and water bodies. The runoff coefficient assigned to different categories ranges from 0 to 1 depending on the land use type as shown in Fig. 3b2. The values were selected on the basis of rational formula for runoff coefficient (Source: http://water.me.vccs.edu/courses/CIV246/table2b.htm).

The net recharge map was derived in GIS using the formula as

netrecharge=precipitation(rainfall)-0.05×precipitation(rainfall)-precipitaion(rainfall)×runoffcoefficients

The net recharge in the thematic map was reclassified into two types and assigned their corresponding ratings. The map layer for net recharge is shown in Fig. 3(b).

### Aquifer media

Aquifer media map was prepared from the geologic map of Ranchi district. Aquifer media in the study area were reclassified into four types and their corresponding ratings were assigned for each aquifer media as given in Table 3. The thematic map is shown in Fig. 3c.

### Soil media

Soil media map was prepared from the soil map of Ranchi district. The soil profile was collected from Birsa Agriculture University (BAU), Ranchi. It was digitized in ArcGIS for generating the thematic map of soil media. The study area consists of fine to coarse loamy-type soil. The soil type was classified into three types and their corresponding ratings were assigned for each type of soil media. The map generated for soil media is shown in Fig. 3d.

### Topography

The topography map was prepared using the shuttle radar topography mission (SRTM) data. The percentage slope raster file was created from Digital Elevation Model (DEM) using spatial analyst. The slope percentage in the study area was reclassified into four classes and assigned their corresponding ratings as given in Table 3. The thematic map layer of topography is shown in Fig. 3e.

### Impact of vadose zone

Due to unavailability of vadose zone data in the study area, information of the soil media was used to derive the approximate ratings for Vadose zone. The map was converted to a raster data by defining ratings for the vadose zone media (using soil media data) (Table 3; Fig. 3d). The thematic map of the impact of vadose zone is shown in Fig. 3f.

### Hydraulic conductivity

Due to unavailability of hydraulic conductivity data in the study area, information of the aquifer media was used to derive the approximate ratings for hydraulic conductivity. It was converted to raster data according to the defined ratings. The ratings of the hydraulic conductivity were assigned (using aquifer media data instead here) as per Table 3. The map of hydraulic conductivity is shown in Fig. 3g.

## Results and discussion

**Fig. 4:**
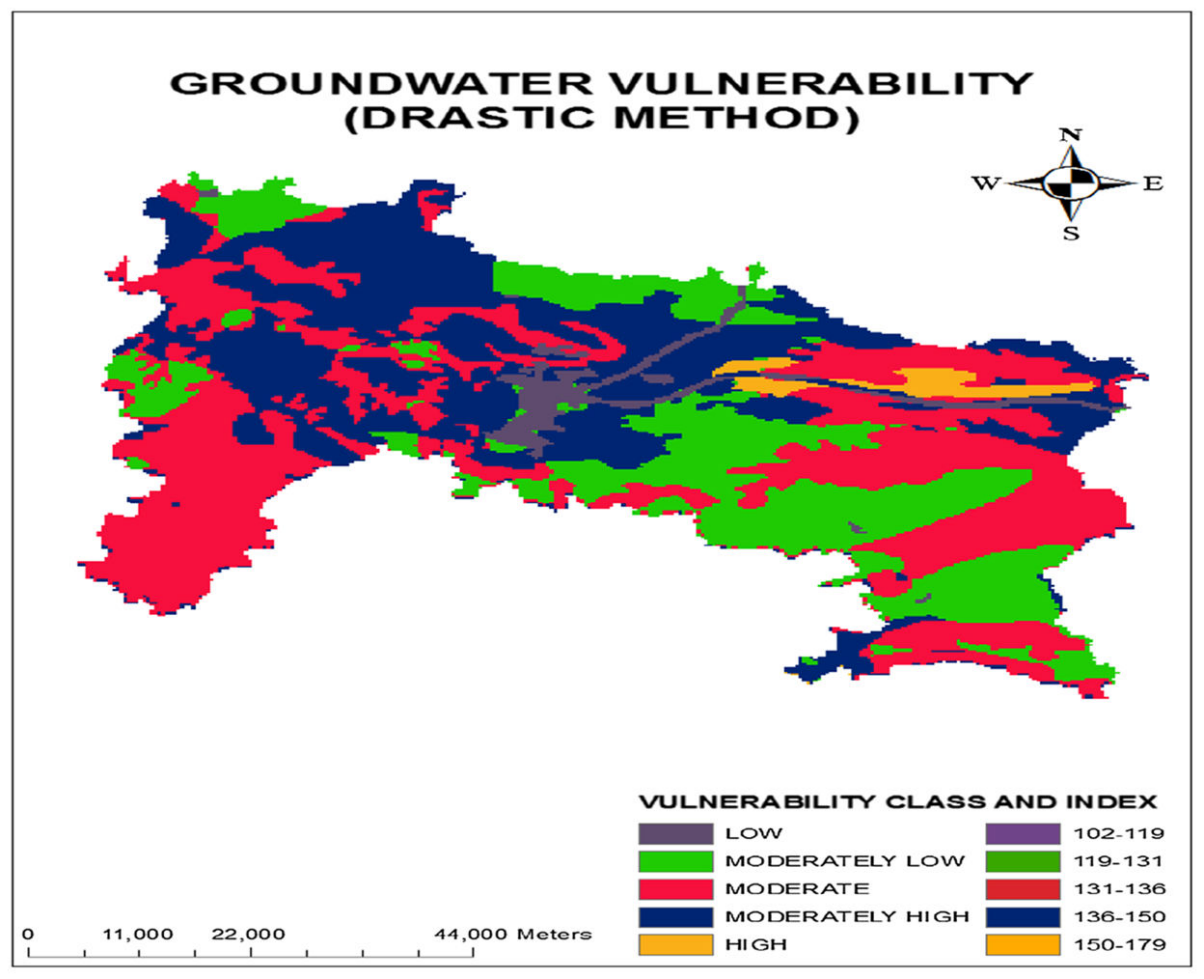
Relative potential of groundwater vulnerability to pollution map

The GIS-based DRASTIC model was developed for generating the aquifer vulnerability map of Ranchi District. This will reflect the aquifer’s inherent capacity to become contaminated. The final map represents the range of the vulnerability indices. The higher the vulnerability index, the higher is the capacity of the hydrogeologic condition to readily move contaminants from surface to the ground-water. On the other hand, low indices represent ground-water is better protected from contaminant leaching by the natural environment. The final vulnerability map was obtained by overlaying the seven hydrogeological thematic layers in ArcGIS software version 9.3. The final ground-water vulnerability map is shown in Fig. 4. The range of the vulnerability indices was reclassified into five classes (low, moderately low, moderate, moderately high, and high) on the basis of Jenks natural breaks that describe the relative probability of contamination of the groundwater resources. A regional scale has been used for comparing the relative vulnerability of groundwater resources.

The result of groundwater vulnerability to pollution assessment indicates that the index value ranged from 102 to 179. The maximum and minimum vulnerability indices were calculated by the sum of the product of maximum and minimum ratings for all the parameters with its corresponding weightage, respectively. The study area was divided into five zones of relative vulnerability: low groundwater vulnerability risk zone (index: 102–119); moderately low vulnerability risk zone (index: 119–131), moderate vulnerability zone (index: 131–136), moderately high vulnerability zone (index: 136–150), and high vulnerability zone (index: 150–179).

The results reveal that the percentage of total area under different vulnerability classes is 3.45 % (168.13 km^2^), 22.12 % (1,075.45 km^2^), 38.85 % (1,890.99 km^2^), 33.63 % (1,636.96 km^2^), and 1.85 % (94.97 km^2^) for low, moderately low, moderate, moderately high and high, respectively. The high vulnerability zones are mainly lie in the blocks of Sonahatu, Angara, and Silli.

$$\begin{array}{l} \text{maximum}\;\text{vulnerability}\;\text{index}=\sum_{i=1}^{7}{\text{rating}}_{i}\times {\text{weightage}}_{i}\\ =10\times 5+9\times 4+10\times 3+10\times 1+10\times 5+10\times 3=226 \end{array}$$

similarly,

$$\begin{array}{l} \text{minimum}\;\text{vulnerability}\;\text{index}=\sum_{i=1}^{7}{\text{rating}}_{i}\times {\text{weightage}}_{i}\\ =1*5+1*4+1*3+1*2+1*1+1*5+1*3=23 \end{array}$$

Though, it is very difficult to say the role of a particular parameter on the spatial changes in the vulnerability index without sensitivity analysis. This is because variation in depth to groundwater table in the study area was found to be low. But the vulnerability map clearly reveals that the depth to groundwater has an insignificant role in spatial changes in vulnerability index. It is clear from the map that the vulnerability is low in the area having higher depth to groundwater and vice versa. Furthermore, the variation of net recharge was very high in the study area and hence had a high influence on the spatial changes in the vulnerability index. Thus, to understand the influence of each parameter sensitivity analysis was carried out. This is explained in sensitivity analysis section.

## Validation of the model

**Fig. 5:**
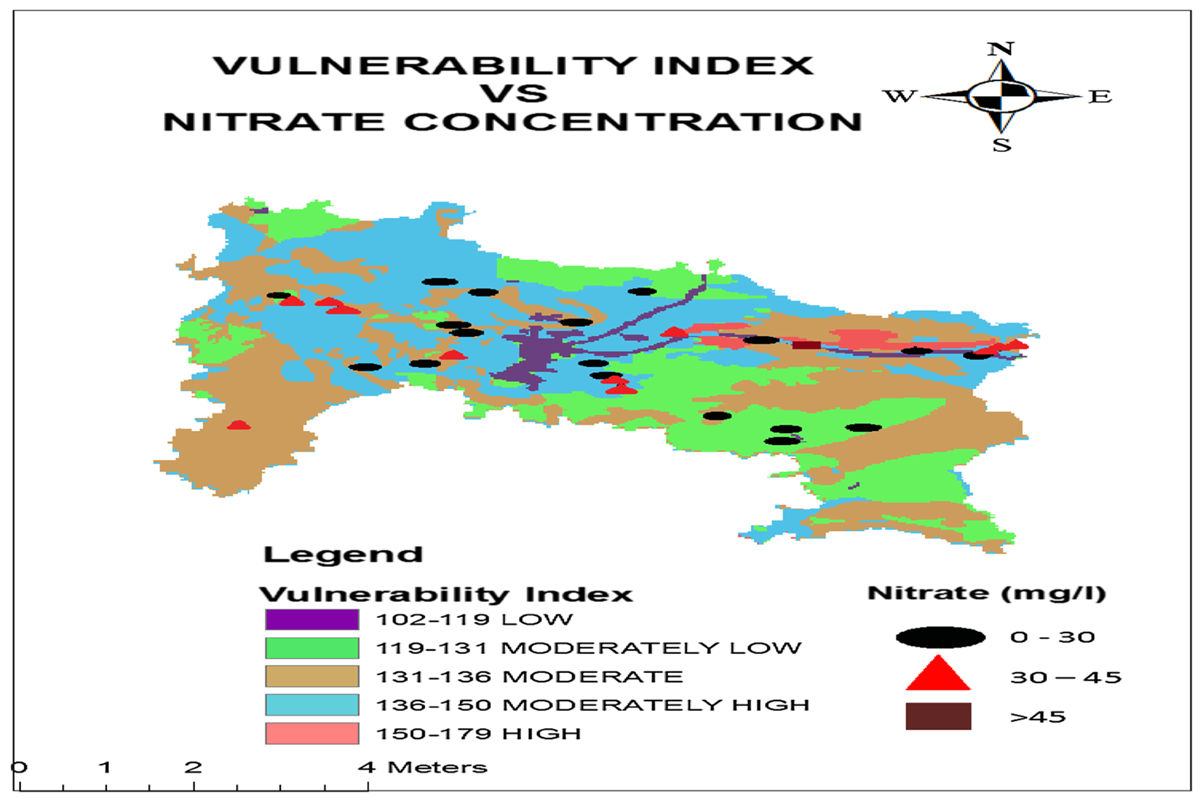
Vulnerability index vs. nitrate concentration

The model was validated by comparing the model output (vulnerability index) with the observed nitrate concentration in groundwater in the study area. The reason behind the selection of nitrate was that the major sources of nitrate in groundwater are various anthropogenic activities like fertilizer used in the agricultural field. The DRASTIC model assumes that the contaminant has the mobility of water. Nitrate being completely soluble in water and hence very nearly satisfies this assumption. Groundwater samples were collected from 30 locations in the study area and analyzed in laboratory for measuring the nitrate concentrations. The spatial locations of the sampling points were recorded by a handheld GPS meter. Nitrate analysis was done as per the standard methods (APHA 1995) using UV–VIS spectrophotometer. In this method generally the absorption was measured twice, i.e., at 220 nm for nitrate concentration and at 275 nm for organic matters which cause hindrance. Then the absorption at 220 nm was subtracted from twice the absorption at 275 nm to obtain the actual nitrate concentration of a given water sample. Nitrate concentrations were found to be in the range of 10.12–51.34 mg/l. The DRASTIC indices of the corresponding points were determined from vulnerability index map (Fig. 4). Figure 5 clearly indicates that the trends of nitrate concentration and vulnerability indices were matched closely in most of the occasions except a few. The correlation between the vulnerability indices and observed nitrate concentration was found to be 0.859.

**Fig. 6:**
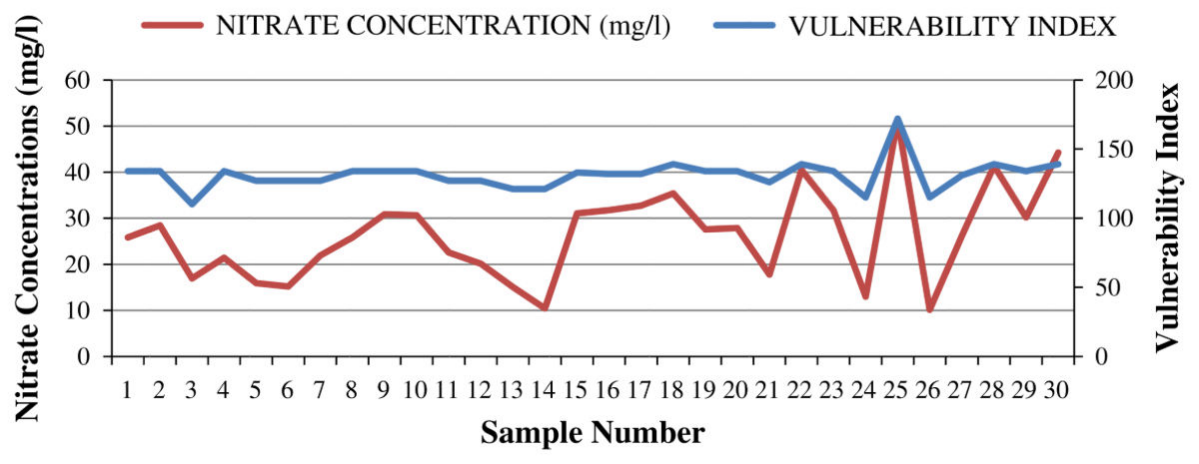
Vulnerability index and corresponding nitrate concentration

This clearly indicates that the model can be accepted for vulnerability assessment and predict with 85.9 percent accuracy. The comparative values of observed nitrate concentration at 30 sampling locations were superimposed on the vulnerability index map as shown in Fig. 6. The range of observed nitrate concentration was classified into three levels (<30 mg/l; 30–45 mg/l; and >45 mg/l) and compared with the corresponding vulnerability indices. Figure 6 clearly indicates that nitrate concentrations of the samples lie down in level 1 (<30 mg/l) were mainly stretched out in the class of moderately low or moderate vulnerability class. Similarly, the nitrate concentration of the samples which lies down in level 2 (30–45 mg/l) was mainly stretched out in the class of moderate or moderately high vulnerability class. It was observed that only one sample had the concentration level greater than 45 mg/l that is in level 3 and this location was stretched out in high vulnerability class. There were few samples that contradicted the law (high vulnerability class zone has high nitrate concentration) due to some other factors which were not defined in the model.

## Sensitivity analysis

The ideas or views of scientists’ conflict in regard to DRASTIC model for groundwater vulnerability to pollution assessment. Some scientists agreed that groundwater vulnerability assessment can be studied without considering all the factors of the DRASTIC model (Merchant 1994), while some others not agreed with the ideas (Napolitano and Fabbri 1996). To make a common consensus sensitivity analysis of the model and groundwater contamination analysis are carried out.

Sensitivity analysis provides information on the influence of rating and weights assigned to each of the factors considered in the model (Gogu and Dassargues 2000b). Lodwik et al. (1990) defined the measure of map removal sensitivity. This explains the degree of sensitivity associated with removing one or more map layers. The sensitivity analysis mentioned above can be measured by removing one or more layer maps using the following equation:

(2) $$S_{i}=\left|\frac{V_{i}}{N}-\frac{V_{xi}}{n}\right|.$$

where, *S_i_* represents sensitivity for *i*th sub-area associated with the removal of one map (*x*-factor), *V_i_* is vulnerability index computed using Eq. (1) for the *i*th sub-area, *V_xi_*, vulnerability index computed for *i*th sub-area excluding o map layer (*x*), *N* is the number of map layers used to compute vulnerability index in Eq. (1) and *n* is number of map layers used for sensitivity analysis. To assess the magnitude of the variation created by removal of one parameter, the variation index was computed as:

(3) $${\text{Var}}_{i}=\left(\frac{V_{i-Vx_{i}}}{V_{i}}\right)\times 100$$

where, Var*_i_* is variation index of the map removal parameter; and *V_i_* and *V_xi_* represent vulnerability index for the *i*th sub-area in two different conditions as mentioned above. Variation index estimates the effect on vulnerability indices due to removal of each parameter. Its value can be either positive or negative, depending on vulnerability index. Variation index directly depends upon the weighting system.

The single parameter sensitivity test was carried out to estimate the role of each parameters considered in the model on the vulnerability measure. The objective of this analysis is to compare the real or “effective” weight of each parameter with that of the corresponding assigned or “theoretical” weight. The effective weight of a parameter in *i*th sub-area can be determined using the following equation:

(4) $$W_{xi}=\left(\frac{X_{ri\times X_{wi}}}{V_{i}}\right)\times 100$$

where, *X*_r_*_i_* and *X*_w_*_i_* represent the rating and the weight assigned to a parameter *X*, respectively, in *i*th sub-area and *V_i_* is the vulnerability index as mentioned above. The sensitivity analysis helps to validate and evaluate the consistency of the analytical results and is the basis for proper evaluation of vulnerability maps. A more efficient interpretation of the vulnerability index can be achieved through sensitivity analysis. The summary of the results of sensitivity analysis that was performed by removing one or more data layer is represented in Tables 4 and 5. Statistical analysis results (shown in Table 4) indicate that the most sensitive to groundwater pollution is the parameter *D*, followed in importance by factors *R*, *I*, *A*, *C*, *S* and *T*. The highest mean value was associated with the depth to groundwater table (4.52) whereas the impact of vadose zone shows the lowest sensitive value (0.32). The results of variation index (shown in Table 5) clearly indicate that the parameter *R* has the highest variation index (0.274) followed by parameter I of variation index (0.234). This variation index explains the effect on vulnerability index on removal of any parameter.

Variation index is directly associated with the weighting system of the model. New or effective weights for each input parameters were computed using the Eqs. (3) and (4) and reported in Table 5. The effective weight factor results clearly indicate that the parameter *D* dominates the vulnerability index with an average weight of 23.84 % against the theoretical weight of 21.74 %. The actual weight of parameter *I* (16.77 %) is smaller than the theoretical weight (21.74). The calculated weight of parameter *T* (7.07 %) is greater than theoretical weight (4.35 %). The highest effective weight of parameter *D* clearly indicates the presence of shallow groundwater table in the most part of the study area and the calculated effective weight of parameter *T* is more than theoretical weight due to the fact that the slope in most of the part of the study area is<6 %.

It is clearly observed in the study that the calculated effective weights for each parameter are not equal to the theoretical weight assigned in DRASTIC method. This is due to the fact that weight factors are strongly related to the value of a single parameter in the context of value chosen for the other parameters. Therefore, the determination of effective weights is very useful to revise the weight factors assigned in DRASTIC method and may be applied more scientifically to address the local issues.

## Conclusions

A GIS-based DRASTIC model was used for computing the groundwater vulnerability to pollution index map of Ranchi district. The study area was divided into five zones (low, moderately low moderate, moderately high and high) on the basis of relative groundwater vulnerability to pollution index. Higher the value of the vulnerability index, higher is the risk of groundwater contamination. The results reveal that moderate vulnerable class covers the maximum percentage of the area (38.85 % of the total area). Moderately high vulnerability class and moderately low vulnerability class also cover significant share of the area.

Sensitivity analysis results indicate that the new effective weights for each parameter are not equal to the theoretical weight assigned in DRASTIC method. Thus, the computation of effective weights is very useful to revise the weight factors assigned in DRASTIC method and may be applied more scientifically to address the local issues.

Groundwater has an important role in drinking water supply in Ranchi district. The study suggests that the GIS-based DRASTIC model can be used for identification of the vulnerable areas for groundwater quality management. In the vulnerable areas, detailed and frequent monitoring of groundwater should be carried out for observing the changing level of pollutants. Furthermore, the present study also helps for screening the site selection for waste dumping.

## Figures and Tables

**Table 1:** Ranges and ratings for various hydrogeological settings (Aller et al. 1987)

Depth to groundwater			Net recharge		
Ranges (m)	Ratings (*D*_r_)	Sub-index (*D*_r_ × *D*_w_)	Ranges (cm)	Ratings (*R*_r_)	Sub-index (*R*_r_ × *R*_w_)
0–1.52	10	50	0–5.08	1	4
1.52–4.57	9	45	5.08–10.16	3	12
4.57–9.14	7	35	10.16–17.78	6	24
9.14–15.24	5	25	17.78–25.4	8	32
15.24–22.86	3	15	25.4+	9	36
22.86–30.48	2	10			
30.48+	1	5			
Weight (*D*_w_)	5		Weight (*R*_w_)	4	
Aquifer type			Soil type		
Type	Ratings (*A*_r_)	Sub-index (*A*_r_ × *A*_w_)	Type	Ratings (*S*_r_)	Sub-index (*S*_r_ × *S*_w_)
Massive shale	2	6	Thin or absent	10	20
Metamorphic/igneous	3	9	Gravel	10	20
Weathered metamorphic/igneous	4	12	Sand	9	18
Glacial till	5	15	Peat	8	16
Bedded sandstone, limestone and shale sequences	6	18	Shrinking and/or aggregated clay	7	14
Massive sandstone	6	18	Sandy loam	6	12
Massive limestone	6	18	Loam	5	10
Sand and gravel	8	24	Silty loam	4	08
Basalt	9	27	Clay loam	3	06
Karst limestone	10	30	Muck	2	04
Weight (*A*_w_)	3		Non-shrinking and non-aggregated clay	1	02
Topography or slope			Weight (*S*_w_)	2	
Ranges (in %)	Ratings (*T*_r_)	Sub-index (*T*_r_ × *T*_w_)	Impact of vadose zone		
0–2	10	10	Type	Ratings (*I*_r_)	Sub-index (*I*_r_ × *I*_w_)
2–6	9	9	Confining layer	1	5
6–12	5	5	Silt/clay	3	15
12–18	3	3	Shale	3	15
18+	1	1	Limestone	6	30
Weight (*T*_w_)	1		Sandstone	6	30
Hydraulic conductivity			Bedded limestone, sandstone, shale	6	30
Range (m/d)	Ratings (*C*_r_)	Sub-index (*C*_w_ × *C*_r_)	Sand and gravel with significant silt and clay	6	30
0.04–4.07	1	3	Metamorphic/igneous	4	20
4.07–12.22	2	06	Sand and gravel	8	40
12.22–28.52	4	12	Basalt	9	45
28.52–40.74	6	18	Karst limestone	10	50
40.74–81.49	8	24			
81.49+	10	30			
Weight (*C*_w_)	3		Weight (*I*_w_)	5	

**Table 2:** Data types and its sources for creation of output layers

Sl. No.	Data types	Sources	Output layer
1	Well data	Real time observation using GPS and tape	Depth to water
2	Average annual rainfall	Indian Meteorological Department, India	Net recharge
3	Geologic map	Central Ground Water Board, PATNA	Aquifer media
4	Soil map	Birsa Agricultural University, RANCHI	Soil media
5	SRTM data	USGS GLOVIS visualization viewer	Topography
6	Soil map	Birsa Agricultural University, RANCHI	Impact of vadose zone
7	Geologic map	Central Ground Water Board, PATNA	Hydraulic conductivity

**Table 3:** Ranges and ratings for various hydrogeological settings using DRASTIC data for the Study area

Sl. No.	Depth to groundwater (ft)	Ratings (*D*_r_)	Net recharge (cm)	Ratings (*R*_r_)	Aquifer media	Rating (*A*_r_)	Soil media	Soil Rating (*S*_r_)	Topography/ slope (in %)	Ratings (*T*_r_)	Impact of vadose zone	Ratings (*I*_r_)	Conductivity (m/d)	Ratings (*C*_r_)
1	6	9	92	9	Weathered metamorphic/igneous	4	Silty loam	4	12	3	Silty loam	4	12.22	4
2	10	9	92	9		4		4	12	3		4	12.22	4
3	9	9	6	3		4		4	12	3		4	12.22	4
4	10	9		9		4		4	12	3		4	12.22	4
5	5	9	91	9		4	Clay loam	3	12	3		4	12.22	4
6	6	9	92	9		4		3	12	3	Clay loam	3	12.22	4
7	5	9	88	9		4		3	12	3		3	12.22	4
8	12	9	90	9		4	Silty loam	4	12	3	Silty loam	4	12.22	4
9	8	9	91	9		4		4	12	3		4	12.22	4
10	9	9	91	9		4		4	12	3		4	12.22	4
11	6	9	90	9		4	Clay loam	3	12	3	Clay loam	3	12.22	4
12	5	9	90	9		4		3	6	5		3	12.22	4
13	5	9	92	9	Bedded sandstone, limestone and shale sequences	5		3	12	3		3	0.04	1
14	4	9	90	9		5		3	19	1		3	0.04	1
15	2	10	88	9		5	Silty loam	4	12	3	Silty loam	4	0.04	1
16	5	10	89	9	Weathered metamorphic/ igneous	4	Clay loam	3	12	3	Clay loam	3	12.22	4
17	2	10	90	9		4		3	12	3		3	12.22	4
18	5	9	91	9		4	Silty loam	4	12	3	Silty loam	4	12.22	4
19	2	9	92	9		4		4	12	3		4	12.22	4
20	2	9	92	9		4		4	6	5		4	12.22	4
21	4	10	92	9	Bedded sandstone, limestone and shale sequences	5	Clay loam	3	6	5	Clay loam	3	0.04	1
22	15	9	91	9	Weathered metamorphic/ igneous	4	Silty loam	4	12	3	Silty loam	4	12.22	4
23	5	9	90	9		4		4	12	3		4	12.22	4
24	2	10	6	3		4		4	12	3		4	12.22	4
25	5	10	90	9	Basalt	9		4	12	3		4	82	10
26	8	10	6	3	Weathered metamorphic/ igneous	4		4	12	3		4	12.22	4
27	5	9	92	9		4	Clay loam	3	12	3	Clay loam	3	12.22	4
28	6	10	6	3		4	Silty loam	4	12	3	Silty loam	4	12.22	4
29	2	9	90	9		4		4	12	3		4	12.22	4
30	5	10	92	9		4		4	12	3		4	12.22	4

**Table 4:** Statistics of single parameter sensitivity analysis

Parameter	Minimum value	Maximum value	Mean	Standard deviation
*D*	3.47	5.71	4.52	0.35
*R*	0.45	3.11	2.71	0.42
*A*	0	1.57	1.09	0.27
*S*	1.21	3.02	1.98	0.19
*T*	1	3.92	2.67	0.23
*I*	0	1.52	0.32	0.28
*C*	0.45	2.92	1.33	0.47

**Table 5:** Assigned weights and effective weights

Parameter	Assigned weight	Assigned weight (%)	Variation index (*W*_x_*_i_*)	Calculated effective weight after rescaling (*X*_w_*_i_*)	Calculated effective weight (%)
*D*	5	21.74	0.26–0.46	6.09–10.65	26.47–46.3
*R*	4	17.39	0.08–0.29	1.89–6.73	8.21–29.27
*A*	3	13.04	0.08–0.19	1.84–4.5	8–19.57
*S*	2	8.69	0.03–0.08	0.79–1.8	3.42–7.84
*T*	1	4.35	0.03–0.08	0.72–1.85	−3.14–8.06
*I*	5	21.74	0.09–0.22	2.06–5.13	8.98–22.32
*C*	3	13.04	0.02–0.22	0.47–5.0	2.06–21.74
